# Consequences of Prematurity on Cortisol Regulation and Adjustment Difficulties: A 9-Year Longitudinal Study

**DOI:** 10.3390/children9010009

**Published:** 2021-12-24

**Authors:** Auriana Urfer, Hélène Turpin, Nevena Dimitrova, Ayala Borghini, Kerstin Jessica Plessen, Mathilde Morisod Harari, Sébastien Urben

**Affiliations:** Division of Child and Adolescent Psychiatry, Lausanne University Hospital (CHUV) and University of Lausanne, 1015 Lausanne, Switzerland; auriana.urfer@unil.ch (A.U.); hm.turpin@gmail.com (H.T.); nevena.e.dimitrova@gmail.com (N.D.); ayala.borghini@bluewin.ch (A.B.); Kerstin.Plessen@chuv.ch (K.J.P.); Mathilde.Morisod@chuv.ch (M.M.H.)

**Keywords:** prematurity, child’s behaviors problems, child’s emotional problems, HPA axis regulation, follow-up

## Abstract

A preterm birth represents a stressful event having potentially negative long-term consequences. Thirty-three children born preterm (<33 weeks gestational age) and eleven full-term children participated in a nine-year longitudinal study. Perinatal Risk Inventory (PERI) was used at birth to assess the perinatal stress. Salivary cortisol, collected four times a day over two consecutive days, was measured with radioimmunoassay technique at six months and nine years to assess the hypothalamic-pituitary-adrenal (HPA) axis. Mothers reported post-traumatic symptoms on a self-report questionnaire 12 months after their child’s birth and children’s adjustment problems at 9 years of child age on the Child Behavior Checklist. Results showed a significant difference in cortisol regulation at nine years between preterm and full-term children but no differences in adjustment problems. Whereas biological factors (i.e., PERI, cortisol regulation at six months) explained cortisol at nine years, maternal post-traumatic symptoms were predictive of adjustment problems in their child. In conclusion, very preterm birth has some long-term consequences on the HPA-axis regulation at nine years. Although cortisol regulation is mostly influenced by biological factors, the presence of maternal post-traumatic symptoms predicts the manifestation of adjustment problems in both groups. This shows the importance of maternal psychological well-being for child development. Further research is needed to understand the exact consequences of premature birth on cortisol regulation and the implication for the child’s development and health.

## 1. Introduction

### 1.1. Prematurity

Preterm birth is defined [1] by birth before 37 weeks of gestation (with sub-categories: moderate preterm—32 to 37 weeks, very preterm—28 to 32 weeks, and extremely preterm—less than 28 weeks). It represented 10% of all living births worldwide [1] and 6.4% in Switzerland, in 2020 [2].

Recent progress in medical techniques and care instructions of preterm infants has enabled to increase the survival rate of premature infants [3,4,5]. However, it is nowadays well established that premature birth has an important impact on the infant and the family not only at birth but also on long-term [6,7]. Indeed, preterm children are at higher risk for long-term medical and developmental difficulties [6,8,9]. In particular, it has been recognized that preterm-born children are more at risk of presenting maladaptive self-regulation skills including stress response system regulation such as the hypothalamic-pituitary-adrenal (HPA) axis [10], as well as the development of behavioral (or externalizing) and emotional (or internalizing) problems [11].

Although some studies have shown that preterm children were more at risk to develop internalizing and externalizing problems [6,7,12], other studies did not observe any difference between preterm and full-term children [13,14]. Internalizing and externalizing problems are often co-morbid and closely related to each other in children and adolescents [15,16,17]. They differ in the way of expressing the distress, either inwardly (i.e., internalizing: depression, anxiety) or outwardly directed (i.e., externalizing: conduct disorder, substance abuse) [18,19]. In our study, we will consider both internalizing and/or externalizing problems and examine them together to define the presence of adjustment problems [20,21].

### 1.2. Preterm Birth: A Stressful Event

Preterm birth can be a particularly stressful event for the newborn and their parents. More specifically, in the context of prematurity, two types of stress might be articulated: perinatal stress (newborn’s stress such as neonatal complications, painful medical procedures) and parental stress (e.g., post-traumatic stress disorder, depression). Early exposure to repeated stress put the preterm-born children at risk for developing behavioral and emotional problems or in other terms, adjustment difficulties [6,11,22]. These difficulties [20,21] often persist into adolescence and early adulthood [23] and can have an important negative impact on their quality of life and their family [24].

#### 1.2.1. Stress Regulation: Hypothalamic-Pituitary-Adrenal (HPA) Axis

From a biological point of view, a stressful event activates the neuro-endocrinologic pathways and more specifically the HPA axis, which results in the release of cortisol [25,26,27]. Beyond the reactivity to a stressful event, the HPA axis also has a circadian rhythm with a high cortisol level in the morning, a gradual decline throughout the day, and the lowest level in the evening [28]. This rhythm is established between three to nine months [29]. However, the exposure to extreme or chronic stress, such as prematurity, can lead to a long-term elevation of cortisol [30,31]. Persistent higher cortisol has been shown to have a toxic effect on neuronal cells, altering the function of different neuronal system, and even affecting brain structures that are essential for self-regulation skills [32,33].

#### 1.2.2. Perinatal Stress

Premature birth is undeniably a stressful event for the newborn. The organism of preterm children is immature and not physiologically ready for an extra-uterine environment [34]. They will spend their first days of life in the neonatal unit, where they might be exposed to several painful and invasive procedures, as well as to sensory dystimulation (i.e., intensive light and noise), in other terms to a highly stressful environment [7,35] which is significantly different from the maternal womb [36]. Moreover, during the neonatal period, neuronal pathways are immature and are therefore particularly vulnerable [37]. Exposition to stress and multisensory dystimulation during this period can induce long term impacts on developmental outcomes, including brain development, stress regulation system and adjustment problems [29,38,39].

#### 1.2.3. Parental Stress

Premature birth is often associated with important parental, and especially maternal stress. Mothers are rapidly separated from their newborn child, and they might have lots of concerns regarding its survival and its medical complications [40,41]. Moreover, preterm birth can be traumatic for mothers and may induce post-traumatic stress disorder (PTSD), depressive symptoms, anxiety, and worry symptoms [42,43]. The presence of maternal stress and PTSD symptoms can affect their emotional and psychological availability for the care of their child and negatively impact the quality of mother-child interactions [44,45].

#### 1.2.4. Impact of Perinatal and Maternal Stress on Child Development

Chronic exposition to stress has been shown to have a detrimental impact on the neuroendocrinological system, as well as on behaviors regulation [46]. Regarding the HPA axis, it is known that early life experience, perinatal stress, and maternal stress can affect the programming of the HPA axis [47,48,49]. Some studies have demonstrated that premature children showed alterations at different levels of the HPA axis. For instance, a study from Grunau et al. has shown that preterm children had a lower level of cortisol at 3 months but a higher level at 8 and 18 months compared to full-term children [50]. Over time, a high level of cortisol may have a detrimental effect on the brain due to the toxic effect on neurons and their myelinization [26,50]. These alterations can influence the development of health problems later in life, such as cardiovascular, metabolic, and psychological problems [48,51].

Moreover, perinatal stress influences the development of brain areas which are important for the regulation of emotions and behaviors [38,52]. Maternal stress has an impact on the mother-child attachment and interactions, which is known to have an important influence on children’s social, cognitive, and emotional development and might later lead to adjustment problems [53,54,55,56].

### 1.3. The Current Study

In summary, a preterm birth is a stressful event for both the child and the parents. It may lead to the development of adjustment problems and dysfunctional HPA axis regulation [27,35,37,47,50]. Although several studies have suggested that children’s development of emotional and behavioral problems and HPA axis regulation were modulated by factors such as perinatal stress and parental attitudes [57,58], the interaction of these factors as a whole is not yet fully understood.

To the best of our knowledge, no longitudinal study examines the influence of perinatal stress and maternal stress on child’s psychophysiological self-regulation skills (i.e., HPA axis regulation and adjustment problems) in the context of intense early stress, namely prematurity. The purpose of the study was to understand the consequences of the stress, induced by prematurity, on the HPA axis regulation and the development of adjustment problems at nine years.

In particular, our primary objective was to examine the interplay between prematurity and HPA axis’ regulation at nine years alongside the presence of adjustment problems. To do so, we will compare preterm to full-term regarding the HPA axis regulation, as well as on adjustment problems. The secondary objective was to specify the risk factors (i.e., perinatal risk, the cortisol level at six months, and the maternal PTSD of the parents) of the HPA axis dysregulation and adjustment problems (i.e., internalizing and/or externalizing symptoms) at nine years. Identifying the risk factors could help to identify risk groups of preterm children that would benefit the most from early intervention.

## 2. Materials and Methods

### 2.1. Participants

All infants (*n* = 242) born before 33 weeks of gestation (very preterm infants -VPT), in the University Hospital of Lausanne between 2005 and 2009 were eligible to participate in the first part of the study. Twenty-nine infants were excluded due to death and an additional eighty-seven parents did not want to participate in the study. The exclusion criteria were parents who were not fluent in French, parental mental illness, infant malformation, periventricular leuko-malacia with a grade > 2 or intraventricular hemorrhage with a grade > 2 and neonatal abstinence syndrome. If an infant was found to have severe mental or psychomotor delays at their six months corrected age (defined as 6 months after 40 weeks of gestational age) pediatric appointment, then the infant was also excluded from the study. Moreover, twenty-five families with an infant born at full-term (gestational age over 37 weeks) were also recruited between 2008 and 2009 in the University Hospital of Lausanne. The exclusion criteria were parents who were not fluent in French, somatic problems, complications during pregnancy or delivery, and parental mental illness.

**Figure 1:**
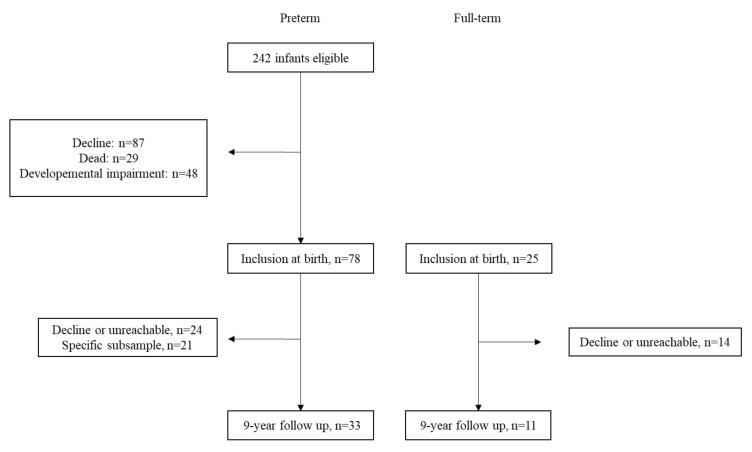
Flow chart.

At the 9-year follow-up, 33 mothers and their preterm children as well as 11 mothers and their full-term children participated in the study. Dropouts were due to non-reachable families or refusal to participate in the follow-up. Moreover, one subsample of preterm children (*n* = 21) did not have the assessment at six months, excluding them from the current study. The Figure 1 illustrates the flow chart.

The data was drawn from an intervention study, where the group of preterm infants were separated into two sub-groups, one with a brief intervention and the other without an intervention. The focus of the intervention was the early relationships between mothers and their child [59]. Since there was no significant difference in terms of cortisol regulation, development of adjustment problems and socio-demographic data, the present study will treat both groups as one sample.

Descriptive statistics are reported in the Table 1.

There was no significant difference between the group of preterm and full-term children in terms of gender (*p* = 0.598), maternal age (*p* = 0.607), and socio-economic status (SES; *p* = 0.418). As expected, the preterm children had a significantly (*p* < 0.001) higher perinatal risk inventory (PERI, see below for details) and a significantly (*p* < 0.001) lower birthweight compared to the full-term children. Dropout analysis showed that there was no significant difference between the group who remained in the study and the participants who dropped out of the study at nine years in terms of gender, parent’s age, socioeconomic status and neonatal data (PERI, birth weight, gestational age). We observed a significant age differences at the follow-up (Preterm: M = 8.99 SD = 0.73, Full-term: M = 9.51, SD = 0.38, *t*(41) = 2.22, *p* = 0.032).

### 2.2. Procedure

**Figure 2:**
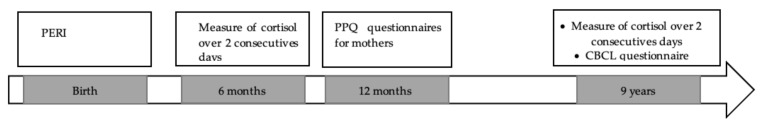
Procedure. Notes. PERI, perinatal risk inventory, PPQ, Perinatal Posttraumatic stress Questionnaire score, CBCL, child behavior checklist.

The design of the study was a longitudinal cohort study (see Figure 2). The study was approved by the Ethics Committee for Clinical Research of the Vaud state (#256/14). Parental written consent was obtained after an exhaustive explanation of the research procedure.

At the birth of the child, various data was collected (e.g., APGAR score, gestational age, birth weight, head growth) to score the PERI [60] and estimate the perinatal risk of the infant.

When the child was six months old (corrected age for premature children), eight “Salivettes” were sent to the parents. The parents were asked to collect the infant’s saliva four times per day over two consecutive days to collect information on the diurnal cortisol profile. Parents also received a leaflet with instructions for the infant’s saliva collection and a diary to write down the time of the collection.

When children were 12 months old, the mothers were asked to rate a PTSD self-questionnaire, the PPQ [61], to evaluate the presence of post-traumatic symptoms linked with the premature birth of their child.

When children were nine years old, families were seen again. They were asked, again, to collect their infant’s saliva four times per over two consecutive days the same way as at six months. Mothers were also asked to fill the child behavior checklist [62], to screen for possible internalizing and externalizing symptoms or in other terms adjustment problems.

### 2.3. Measures

#### 2.3.1. Socio-Economic Status

Socio-economic status (SES) was assessed by the use of an adaptation of the Hollingshead index [63]. This index measures the social status of an individual (SES) and is based on the average of four scores: the maternal and paternal education level and professional occupation rated each on a four-point scale (i.e., degree: 1 = compulsory school, 4 = university grade completed; occupation: 1 = no job/unqualified employee, 4 = senior banker or physician in a private practice). A higher score means higher SES.

#### 2.3.2. Perinatal Risk Inventory: PERI

The perinatal risk inventory (PERI) [60] is an 18-item inventory used to describe the severity of the infant’s perinatal problems. Each item is scored from zero to three and includes the APGAR score, the gestational age, birth weight, head circumference, the presence or absence of congenital infection, the absence or presence of seizures, the nature of the electroencephalogram, cranial computed tomographic, ultrasound, presence of sepsis and/or meningitis, the duration of ventilation, presence of polycythemia, hypoglycemia, hyperbilirubinemia, and long-term physical disabilities. The total score is obtained by adding up all of the items. The PERI was used to estimate the perinatal risk of each infant, with a higher score reflecting higher perinatal risk [60]. It is also correlated with the duration of the hospital stay and intensive procedures, giving an indication of the stress experienced by the preterm child.

#### 2.3.3. Cortisol Regulation

The diurnal cortisol measure was collected over two consecutive days at home totaling four saliva samples per day: just after waking up (08:00), before mealtime at noon (12:00), in the afternoon (17:00), and in the evening (20:00).

In cases of infection, fever or antibiotic treatment, the procedure was delayed. Cotton rolls containing saliva were sent by post to the lab of Prof. Kirschbaum [64] from the Technical University of Dresden (Germany) for cortisol concentration measurements by enzyme immunoassay. Cortisol concentrations were expressed as nanomoles per liter (nmol/L). As a standard procedure [59], the raw cortisol levels received from the lab were transformed into logarithms (log10) allowing the approximation of the Gaussian-like distribution.

We computed the area under the curve with respect to the ground (AUC_G_) with our cortisol measures to estimate ultradian and circadian changes of hormones and to assess the overall secretion over the day. The AUC_G_ represents the total hormonal output.

We used one deriving formula for computation of the AUC_G_:

$$AUC_{G}\;=\;\overset{n-1}{\underset{i=1}{\sum }}\frac{\left(m_{\left(i+1\right)}+m_{i}\right)\;\cdot \;t_{i}}{2}$$

With *t_i_* denoting the individual time distance between measurements, *m_i_* the individual measurements, and *n* the total amount of measurement [65].

#### 2.3.4. Maternal Post-Traumatic Stress Symptoms

Mothers were asked to fill out a self-report questionnaire about post-traumatic stress symptoms, the Perinatal Post-Traumatic Stress Disorders Questionnaire (PPQ) [61], when their child was 12 months old. The PPQ is a 14 questions questionnaire based on the Diagnostic and Statistical Manual of Mental Disorders IV criteria which assesses maternal symptoms of PTSD related to the infant’s perinatal period. Questions are retrospective, mothers are asked to answer about situations they have experienced since and during the six months following their childbirth, and which lasted more than one month. Higher scores mean higher PTSD symptoms. 

#### 2.3.5. Adjustment Problems

The Child Behavior Checklist (CBCL) [62] was used to detect behavioral and emotional problems in children and adolescents from 6 to 18 years of age. It consists of 113 questions answered by the parents and scored on a three-point Likert scale (0 = absent, 1 = occurs sometimes, 2 = occurs often). These scales have good internal reliability (α_s_ > 0.80), with relatively high 1-week test–retest reliability (*r_s_* > 0.85) reported [62,66]. Adjustment problems were computed by averaging externalizing and internalizing T-scores [20,21]. Higher scores refer to more adjustment problems.

### 2.4. Data Analysis

The data were analyzed with SPSS version 27. As a first step, we examined the differences between preterm and full-term infants on neuroendocrine regulation by computing a repeated-measures (four measures a day) analysis of covariance (RM-ANCOVA) by groups (full-term vs. preterm) controlling for age differences whereas ANCOVAs was computed to assess the between-group differences in adjustment problems and AUC_G_. Then, we computed Bravais-Pearson coefficients of correlations between PERI, cortisol regulation (i.e., AUC_G_, maternal PTSD and adjustment problems). Finally, we used two separate regression models to identify possible predictors (i.e., PERI, early cortisol regulation, maternal PTSD) of children adjustment problems (i.e., externalizing and/or internalizing symptoms) or cortisol regulation (i.e., AUC_G_) at nine years. Regarding the important collinearity between group and PERI, we used only PERI scores in the regression models. Age was also introduced in the model as covariate. 

## 3. Results

### 3.1. Between Group Differences on Neuroendocrine Regulation and Adjustment Problems

Descriptive data are reported in Table 2.

Preterm showed higher AUC_G_ at nine years of age compared to full-term children. By contrast, the ANCOVA comparing the group on adjustment problems revealed no difference.

**Figure 3:**
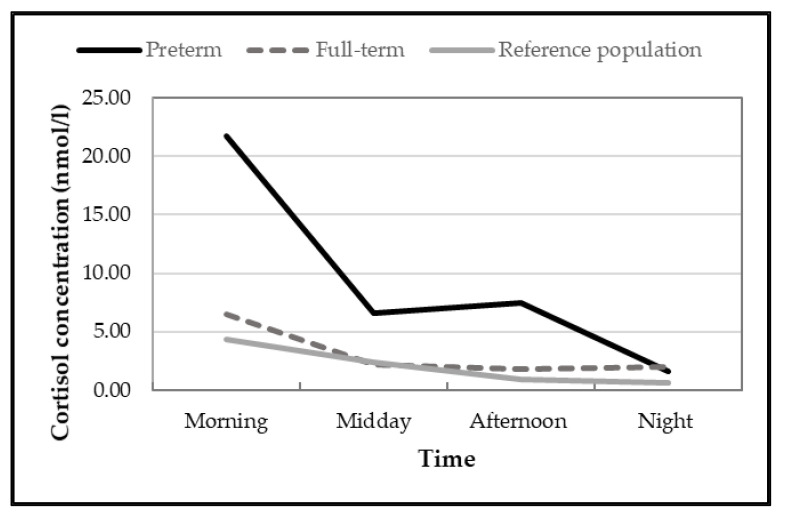
Mean cortisol levels over two days at nine years. Notes. Reference population, reference range values from the CIRCORT database meta-analysis [67].

Figure 3 shows the diurnal cortisol evolution by groups. 

The RM-ANCOVA on the cortisol data revealed no main effect of time, a main effect of group, (*F*(1, 30) = 9.44, *p* = 0.004 ɳ_2_*^p^* = 0.239). A significant interaction effect between time and group (*F*(3, 90) = 4.91, *p* < 0.01, ɳ_2_*^p^* = 0.141) was also observed. In particular, post hoc pairwise comparisons indicated that morning and midday results showed that preterm children had significantly higher cortisol levels than full-term children (morning: *p* = 0.001; midday: *p* = 0.004). In the afternoon and at night, cortisol levels did not differ between preterm and full-term. In the preterm group, morning cortisol levels were significantly higher than other measures (*ps* < 0.001). There was a significant difference between levels at midday and in the evening (*p* < 0.001). At night, cortisol levels were significantly lower than in the afternoon (*p* = 0.012). In the full-term group, there was no significant differences between the daytime.

Finally, we compared our results to reference values from a meta-analysis from Miller et al. [68]. Our group control was small (*n* = 11) and we wanted to enforce our results by comparing with reference values. We observed that our full-term children did not differ significantly from reference values, except for the afternoon data (*p* = 0.005). However, preterm values were significantly higher than reference values for all of the values throughout the day (*ps* < 0.002).

### 3.2. Association between Neuro-Endocrine Regulation, Adjustment Problems and Stress

Table 3 describes the correlations analysis. We can observe that a higher PERI is related to a higher AUC_G_ at nine years. Moreover, maternal PTSD is linked to a lower AUC_G_ at six months, and higher adjustment problems.

The regression analysis conducted on AUC_G_ at nine years revealed that predictors (i.e., PERI, maternal PTSD, AUC_G_ at six months) explain a significant part of the variance, *F*(5, 29) = 5.46, *p* = 0.002, *R*^2^ = 0.532. In particular, higher AUC_G_ at nine years is predicted by a higher PERI and lower adjustment problems. In addition, the regression analysis on adjustment problems revealed that the predictors explain a significant part of the variance, *F*(5, 29) = 2.94, *p* = 0.033, *R*^2^ = 0.380. In particular, higher adjustment problems are related to higher maternal PTSD and a lower AUC_G_ at nine years (Table 4).

## 4. Discussion

The purpose of the present study was to analyze the consequences of stress related to a preterm birth on the child’s HPA axis regulation, as well as on the development of adjustment problems at nine years. We also examined which factors predict the self-regulatory outcomes at nine years (i.e., HPA regulation and adjustment problems) to identify high risk populations where preventive intervention could play an important role. In particular, this study highlighted a significant difference in cortisol regulation at nine years between preterm and full-term children, and showed that the biological stress (i.e., PERI and cortisol at six months) explains the diurnal cortisol concentration at nine years. Age is related to AUC_G_. We did not find any significant differences in adjustment problems in general between the two groups. However, we could show that maternal PTSD’s symptoms at 12 months (i.e., maternal stress) were predictive of adjustment problems in their child at 9 years. We did also show that higher adjustment problems were related to lower AUC_G_ at nine years, which is in line with previous studies [68,69,70]. 

### 4.1. Cortisol Regulation

The main between-group difference lay in the morning cortisol levels. Indeed, preterm children had a higher cortisol level in the morning compared to full-term ones, as well as a higher AUC_G_. Likewise, De Graaf et al. [71] showed that at five years, preterm children had higher cortisol levels in comparison to full-term children. Grunau et al. [50] also reported greater levels of cortisol at 8 months and 18 months in preterm children. A possible explanation for these high levels is that premature birth impacts the early programming of the HPA axis, which was shown in the animal model [48,72,73], leading to long-term consequences. In humans, the development of the neuroendocrine regulation occurs in the postnatal period. This period is characterized by elevated synaptic plasticity, making the brain particularly vulnerable and sensitive [74]. Adverse events such as a premature birth can have an impact on the development and the “programming” of the HPA axis. This study is the first one identifying consequences on the HPA regulation nine years later.

Moreover, we have shown that the level of stress endured by the preterm infants (i.e., PERI) was related to higher cortisol release at nine years (i.e., AUC_G_). This suggests that more stress during the neonatal period contributes to more alteration of the HPA axis regulation up to nine years. This might be related to the impact of stress on several brain structures (i.e., cortex, thalamus, basal ganglia) following a premature birth [38,75,76]. These brains areas are responsible for the neuroendocrine regulation systems such as the HPA axis [38].

### 4.2. Adjustment Problems

We did not find between-group differences regarding adjustment problems. Although some previous studies have demonstrated that preterm children were more at risk to develop adjustment problems [6,7,24,77,78], others have found no differences between late preterm and full-term infants at a young age [13,14]. Our findings can be explained by the fact that the children with biomedical complications (i.e., leukomalacia, intraventricular hemorrhage, mental or psychomotor delay at 6six months) were excluded from our study, whereas various other studies included preterm children with neurological damage [7,79]. Our sample may therefore be considered as low risk of developing adjustment problems and our results seem to indicate that children without biomedical complications are not more at risk to develop adjustment problems. 

The level of stress at birth (i.e., PERI) was not associated with the presence of adjustment problems in the child, which is in line with a previous studies [80]. This emphasizes the fact that other factors play a key role in the development of these problems later in childhood. Indeed, the quality of the postnatal environment (i.e., parental interaction, parental stress) has been proved to have a higher impact on the development of adjustment problems than the somatic risk (i.e., neonatal variables: low birth weight, gestational age) [7,81]. Our results further emphasize that the presence of maternal PTSD symptoms was associated with adjustment problems in the child. There are many ways in which PTSD might affect a child’s development. The presence of PTSD might have an impact on the capacity of mothers to understand their infant’s needs and signals [82], and thus tend to be less sensitive towards their infant [43,44,80,83]. PTSD therefore has an impact both on the mother’s role and the mother-child interaction. The mother-infant attachment, which is the relationship that develops between an infant and its caregiver early in infancy [84], is also important to understand how maternal stress and PTSD can influence the child’s development. Indeed, a secure attachment is essential for optimal child development. The presence of mother PTSD symptoms may lead to an insecure attachment, which has been shown to be associated with greater mental health problems [84,85]. Therefore, symptoms of maternal PTSD, by affecting attachment relationships, can explain the risk of children developing adjustment problems [86].

### 4.3. Clinical Implications

One of our objectives was to identify a high-risk population that would benefit the most from early interventions. We have shown that the presence of postnatal PTSD symptomatology in the mother was a risk factor for the child to develop adjustment problems. This result emphasizes the importance of considering the mother’s mental well-being in the child development. More attention should be given to children whose mothers experience PTSD symptoms following premature birth. The development of preventive intervention concerning specifically maternal PTSD symptoms due to preterm birth could be beneficial for the child’s development and health. Early interventions enhancing the mother-child relationship seem to have a positive effect on child development [87]. It would be also important to develop interventions focusing specifically on maternal PTSD symptoms. Since PTSD symptoms have been shown to affect the quality of the mother-child interaction [83], reducing these symptoms may be a way to improve the quality of this relationship and have a positive impact on the preterm development [88,89]. However, studies focusing on PTSD are limited [90], and future research is needed to develop interventions and evaluate their impact. In the neonatal unit, mental health professionals could offer the possibility to parents to talk about their experience and express their intense emotions, as well as joint observation to help understand and interpret their child’s behavior. This could reinforce their investment in their child and mother-child interaction, which might have a positive effect on child outcomes [44,83,91].

Another important finding was the presence of persistent alterations of the HPA axis regulation even after nine years, which seems related to the severity of the perinatal stress. Early intervention could focus on the reduction of infant’s stress, for instance through skin-to-skin contact (kangaroo care), which has been shown to reduce stress reactivity [92,93], cortisol level at short term [94,95], and improve brain development [96] in preterm children. However, it is not known if this method has a long-term effect on cortisol regulation. Kangaroo care also has positive effects on attachment and on parental stress levels. This might, in turn, have positive effects on the preterm development of the stress regulation system [92,97]. Interventions in the neonatal unit environment could also be offered, for instance by limiting the exposure to deleterious stimulations (i.e., painful procedures, noise) or by adapting the different sensorial stimulations (i.e., auditory, tactile, visual and vestibular) to support the neonate’s brain development and stress regulation [91,98,99]. 

### 4.4. Limitations and Strengths

The present study has some limitations. First, the size of our sample was relatively small. Although we had access to longitudinal information from the birth to nine years, no information was available on events between six months and nine years (i.e., traumatic event, parental separation). Additionally, the study relied only on the mother’s perspective for the assessment of the children’s behavioral problems (CBCL). Results could be influenced by the mental health of the mother. The teacher’s and father’s perspectives for instance would have been beneficial for a more comprehensive view of the children’s adjustment problems, as well as using other questionnaires. Finally, although the PPQ has good reliability [61], it was retrospective rated and self-reported.

The strengths of this study are, first, to have a longitudinal information from birth to nine years. Moreover, we articulated the biological and psychological aspects of stress due to prematurity on the long-term outcomes of the children. 

## 5. Conclusions

This study highlights that very preterm birth has long-term consequences on the cortisol regulation at nine years and that it is mostly influenced by perinatal stress (i.e., PERI). We show that the presence of maternal PSTSD’s symptoms contrary to biological factors is a predictor of the development of adjustment problems, underlying the importance of maternal psychological well-being and the negative impact of maternal PTSD symptoms on the child’s adjustment development. The most important clinical implications should be to develop preventive intervention dealing with postnatal stress and maternal PTSD symptoms, which would be beneficial for the child development and health. 

Finally, the association between cortisol regulation and adjustment problems is complex. The question of whether dysregulation of cortisol is one of the mechanisms putting preterm children more at risk for the development of adjustment problems later in life still needs to be elucidated. Further works are needed to have a better understanding of the detrimental effect of postnatal stress, including perinatal and maternal stress, on the later development of preterm born children. It would be beneficial for the development of targeted and individualized intervention. 

## Figures and Tables

**Table 1:** Socio-demographic variables and dropout analysis.

	Included (N = 44)		Dropout (N = 38)		*p* ^1^	*p* ^2^	*p* ^3^
	1. Preterm	2. Full-Term	3. Preterm	4. Full-Term			
	*n* = 33	*n* = 11	*n* = 24	*n* = 14			
GA	30.11 (1.84)	40.18 (0.90)	30.15 (2.00)	39.34 (1.49)	0.001	ns	ns
Gender (F/M)	54.5/45.5	36.4/63.6	37.5/62.5	35.7/64.3	ns	ns	ns
SES	2.71 (0.61)	2.55 (0.47)	2.54 (0.88)	2.56 (0.93)	ns	ns	ns
Mat. Age (yrs)	32.73 (4.30)	31.64 (5.08)	33.75 (4.19)	30.86 (5.17)	ns	ns	ns
PERI	7.73 (2.38)	0.00 (0.00)	8.08 (3.45)	0.00 (0.00)	<0.001	ns	ns
BW (gr)	1369.24 (373.63)	3383.00 (425.02)	1406.46 (418.73)	3256.43 (365.36)	<0.001	ns	ns
PPQ	2.73 (2.67)	1.22 (1.64)	3.56 (3.31)	0.90 (1.45)	ns	ns	ns

Data expressed in Mean (SD) except for gender. ^1^ *p*-value of the results of *t*-tests comparison between the preterm and full-term included in the follow-up. ^2^ *p*-value of the results of *t*-tests comparison between the 2 group of preterm (drop out vs. included). ^3^ *p*-value of the results of *t*-tests comparison between the 2 groups of full-term (drop out vs. included). Notes. GA, gestational age; F, female; M, male; SES, socio-economic status; Mat., maternal; PERI, perinatal risk inventory score; BW, birth weight; PPQ, Perinatal Posttraumatic stress Questionnaire score.

**Table 2:** Descriptive data on cortisol regulation and adjustment problems in VPT and FT children.

	Preterm (*n* = 33)	Full-Term (*n* = 11)	*p* ^1^	ɳ_2_^p^
AUC_G_	106.51 (42.14)	35.39 (17.94)	<0.001	0.34
Adjustment problems	52.63 (7.04)	53.09 (6.93)	*ns*	*-*

Data expressed in Mean (SD). ^1^ *p*-value of the results of ANCOVA between the two groups and controlling for age differences. Notes. AUC_G_, area under the curve with respect to the ground.

**Table 3:** Bravais-Pearson coefficient of correlations.

	PERI	Maternal PPQ	AUC_G_ at 9y	AUC_G_ at 6m
Maternal PTSD	0.29			
AUC_G_ at 9y	0.53 **	0.22		
AUC_G_ at 6m	−0.26	−0.37 *	−0.17	
Adjustment problems	0.05	0.41 **	−0.25	0.04

* *p* < 0.05, ** *p* < 0.01. Notes. AUC_G_, area under the curve with respect to the ground; PERI: perinatal risk inventory; PPQ, Perinatal Posttraumatic Stress Questionnaire score.

**Table 4:** Regression analysis explaining cortisol regulation (AUC_G)_ or adjustment problems at nine years.

Criterion	Variables	*β*	*p*
AUC_G_ at 9y	Age	−0.364	0.022
	PERI	0.418	0.012
	Maternal PPQ	0.271	0.137
	Adjustment problems	−0.414	0.014
	AUC_G_ at 6m	0.136	0.403
Adjustment problems	Age	−0.222	0.245
	PERI	0.236	0.243
	Maternal PPQ	0.522	0.009
	AUC_G_ 9y	−0.549	0.014
	AUC_G_ at 6m	0.232	0.212

Notes. AUC_G_, area under the curve with respect to the ground; PERI: perinatal risk inventory; PPQ, Perinatal Posttraumatic Stress Questionnaire score.

## Data Availability

Data might be shared upon demands.

## References

[1] World Health Organization (2018). Preterm Birth: Fact Sheet.

[2] Office Fédéral de la Statistique (2020). Santé Des Nouveau-Nés.

[3] Cormack B.E., Harding J.E., Miller S.P., Bloomfield F.H. (2019). The influence of early nutrition on brain growth and neurodevelopment in extremely preterm babies: A narrative review. Nutrients.

[4] Hack M., Fanaroff A.A. (2000). Outcomes of children of extremely low birthweight and gestational age in the 1990s. Semin. Neonatol..

[5] Kumar R.K., Singhal A., Vaidya U., Banerjee S., Anwar F., Rao S. (2017). Optimizing Nutrition in Preterm Low Birth Weight Infants—Consensus Summary. Front. Nutr..

[6] Bhutta A.T., Cleves M.A., Casey P.H., Cradock M.M., Anand K.J.S. (2002). Cognitive and Behavioral Outcomes of School-Aged Children Who Were Born Preterm. JAMA.

[7] Cassiano R.G.M., Gaspardo C.M., Linhares M.B.M. (2016). Prematurity, neonatal health status, and later child behavioral/emotional problems: A systematic review. Infant Ment. Health J..

[8] Luu T.M., Katz S.L., Leeson P., Thébaud B., Nuyt A.M. (2016). Preterm birth: Risk factor for early-onset chronic diseases. CMAJ.

[9] Woodward L.J., Moor S., Hood K.M., Champion P.R., Foster-Cohen S., Inder T.E., Austin N.C. (2009). Very preterm children show impairments across multiple neurodevelopmental domains by age 4 years. Arch. Dis. Child. Fetal Neonatal Ed..

[10] Blair C. (2010). Stress and the Development of Self-Regulation in Context. Child Dev. Perspect..

[11] Chapieski M.L., Evankovich K.D. (1997). Behavioral effects of prematurity. Semin. Perinatol..

[12] Treyvaud K., Ure A., Doyle L.W., Lee K.J., Rogers C.E., Kidokoro H., Inder T.E., Anderson P.J. (2013). Psychiatric outcomes at age seven for very preterm children: Rates and predictors. J. Child Psychol. Psychiatry Allied Discip..

[13] Pérez-Pereira M., Baños L. (2019). Do healthy preterm children have behavior problems?. An. Psicol..

[14] Stene-Larsen K., Lang A.M., Landolt M.A., Latal B., Vollrath M.E. (2016). Emotional and behavioral problems in late preterm and early term births: Outcomes at child age 36 months. BMC Pediatr..

[15] Achenbach T.M., Howell C.T., Quay H.C., Conners C.K. (1991). National survey of problems and competencies among four- to sixteen-year-olds: Parents’ reports for normative and clinical samples. Monogr. Soc. Res. Child Dev..

[16] Lilienfeld S.O. (2003). Comorbidity between and within childhood externalizing and internalizing disorders: Reflections and directions. J. Abnorm. Child Psychol..

[17] White B.A., Jarrett M.A., Ollendick T.H. (2013). Self-regulation deficits explain the link between reactive aggression and internalizing and externalizing behavior problems in children. J. Psychopathol. Behav. Assess..

[18] Carragher N., Krueger R.F., Eaton N.R., Slade T. (2015). Disorders without borders: Current and future directions in the meta-structure of mental disorders. Soc. Psychiatry Psychiatr. Epidemiol..

[19] Willner C.J., Gatzke-Kopp L.M., Bray B.C. (2016). The dynamics of internalizing and externalizing comorbidity across the early school years. Dev. Psychopathol..

[20] Donado C., Friedrich Y., Kossowsky J., Locher C., Koechlin H. (2020). Exposure to Parental Depressive Symptoms: A Longitudinal Analysis on the Association with Adolescents’ Depressive Symptoms and Adjustment Problems. J. Dev. Behav. Pediatr..

[21] Koechlin H., Donado C., Berde C.B., Kossowsky J. (2018). Effects of Childhood Life Events on Adjustment Problems in Adolescence: A Longitudinal Study. J. Dev. Behav. Pediatr..

[22] Dimitrova N., Turpin H., Borghini A., Harari M.M., Urben S., Muller-Nix C. (2018). Perinatal stress moderates the link between early and later emotional skills in very preterm-born children: An 11-year-long longitudinal study. Early Hum. Dev..

[23] Cooke R.W.I. (2004). Health, lifestyle, and quality of life for young adults born very preterm. Arch. Dis. Child..

[24] Hayes B., Sharif F. (2009). Behavioural and emotional outcome of very low birth weight infants- literature review. J. Matern. Fetal Neonatal Med..

[25] Charmandari E., Tsigos C., Chrousos G. (2005). Endocrinology of the Stress Response. Annu. Rev. Physiol..

[26] Sapolsky R.M., Romero L.M., Munck A.U. (2000). How do glucocorticoids influence stress responses? Integrating permissive, suppressive, stimulatory, and preparative actions. Endocr. Rev..

[27] Smith S.M., Vale W.W. (2006). The role of the hypothalamic-pituitary-adrenal axis in neuroendocrine responses to stress. Dialogues Clin. Neurosci..

[28] Guilliams T.G., Edwards L. (2010). Chronic stress and the HPA axis: Clinical assessment and therapeutic considerations. Standard.

[29] Van Bodegom M., Homberg J.R., Henckens M.J.A.G. (2017). Modulation of the hypothalamic-pituitary-adrenal axis by early life stress exposure. Front. Cell. Neurosci..

[30] Faravelli C. (2012). Childhood stressful events, HPA axis and anxiety disorders. World J. Psychiatry.

[31] Ng P.C. (2000). The fetal and neonatal hypothalamic-pituitary-adrenal axis. Arch. Dis. Child. Fetal Neonatal Ed..

[32] Lupien S.J., de Leon M., de Santi S., Convit A., Tarshish C., Nair N.P., Thakur M., McEwen B.S., Hauger R.L., Meaney M.J. (1998). Cortisol levels during human aging predict hippocampal atrophy and memory deficits. Nat. Neurosci..

[33] Lupien S.J., McEwen B.S., Gunnar M.R., Heim C. (2009). Effects of stress throughout the lifespan on the brain, behaviour and cognition. Nat. Rev. Neurosci..

[34] Maitre N.L., Key A.P., Chorna O.D., Slaughter J.C., Matusz P.J., Wallace M.T., Murray M.M. (2017). The Dual Nature of Early-Life Experience on Somatosensory Processing in the Human Infant Brain. Curr. Biol..

[35] Grunau R.E., Holsti L., Peters J.W.B. (2006). Long-term consequences of pain in human neonates. Semin. Fetal Neonatal Med..

[36] El-Metwally D.E., Medina A.E. (2020). The potential effects of NICU environment and multisensory stimulation in prematurity. Pediatric Res..

[37] Graham Y.P., Heim C., Goodman S.H., Miller A.H., Nemeroff C.B. (1999). The effects of neonatal stress on brain development: Implications for psychopathology. Dev. Psychopathol..

[38] Lammertink F., Vinkers C.H., Tataranno M.L., Benders M.J.N.L. (2021). Premature Birth and Developmental Programming: Mechanisms of Resilience and Vulnerability. Front. Psychiatry.

[39] Sullivan M.C., Winchester S.B., Bryce C.I., Granger D.A. (2017). Prematurity and perinatal adversity effects hypothalamic-pituitary-adrenal axis reactivity to social evaluative threat in adulthood. Dev. Psychobiol..

[40] Ionio C., Mascheroni E., Colombo C., Castoldi F., Lista G. (2019). Stress and feelings in mothers and fathers in NICU: Identifying risk factors for early interventions. Prim. Heal. Care Res. Dev..

[41] Pierrehumbert B., Nicole A., Muller-Nix C., Forcada-Guex M., Ansermet F. (2003). Parental post-traumatic reactions after premature birth: Implications for sleeping and eating problems in the infant. Arch. Dis. Child. Fetal Neonatal Ed..

[42] Barkmann C., Helle N., Bindt C. (2018). Is very low infant birth weight a predictor for a five-year course of depression in parents? A latent growth curve model. J. Affect. Disord..

[43] Treyvaud K., Lee K.J., Doyle L.W., Anderson P.J. (2014). Very preterm birth influences parental mental health and family outcomes seven years after birth. J. Pediatr..

[44] Forcada-Guex M., Borghini A., Pierrehumbert B., Ansermet F., Muller-Nix C. (2011). Prematurity, maternal posttraumatic stress and consequences on the mother-infant relationship. Early Hum. Dev..

[45] Hagen I.H., Iversen V.C., Svindseth M.F. (2016). Differences and similarities between mothers and fathers of premature children: A qualitative study of parents’ coping experiences in a neonatal intensive care unit. BMC Pediatr..

[46] Finken M.J.J., Van Der Voorn B., Hollanders J.J., Ruys C.A., De Waard M., Van Goudoever J.B., Rotteveel J. (2017). Programming of the Hypothalamus-Pituitary-Adrenal Axis by Very Preterm Birth. Ann. Nutr. Metab..

[47] Habersaat S., Borghini A., Nessi J., Forcada-Guex M., Müller-Nix C., Pierrehumbert B., Ansermet F. (2014). Effects of Perinatal Stress and Maternal Traumatic Stress on the Cortisol Regulation of Preterm Infants. J. Trauma. Stress.

[48] Matthews S. (2002). Early programming of the hypothalamo–pituitary–adrenal axis. Trends Endocrinol. Metab..

[49] Philbrook L.E., Hozella A.C., Kim B.R., Jian N., Shimizu M., Teti D.M. (2014). Maternal emotional availability at bedtime and infant cortisol at 1 and 3months. Early Hum. Dev..

[50] Grunau R.E., Haley D.W., Whitfield M.F., Weinberg J., Yu W., Thiessen P. (2007). Altered Basal Cortisol Levels at 3, 6, 8 and 18 Months in Infants Born at Extremely Low Gestational Age. J. Pediatr..

[51] McEwen B.S. (1999). Stress and hippocampal plasticity. Annu. Rev. Neurosci..

[52] Lengua L.J., Zalewski M., Fisher P., Moran L. (2013). Does HPA-Axis Dysregulation Account for the Effects of Income on Effortful Control and Adjustment in Preschool Children?. Infant Child Dev..

[53] Bozkurt O., Eras Z., Sari F.N., Dizdar E.A., Uras N., Canpolat F.E., Oguz S.S. (2017). Does maternal psychological distress affect neurodevelopmental outcomes of preterm infants at a gestational age of ≤32 weeks. Early Hum. Dev..

[54] Brumariu L.E., Kerns K.A. (2010). Parent-child attachment and internalizing symptoms in childhood and adolescence: A review of empirical findings and future directions. Dev. Psychopathol..

[55] Pisoni C., Garofoli F., Baiardini I., Tzialla C., Stronati M. (2014). The development of parents-infant relationship in high-risk pregnancies and preterm birth. J. Pediatric Neonatal Individ. Med..

[56] Turpin H., Urben S., Ansermet F., Borghini A., Murray M.M., Muller-Nix C. (2019). The interplay between prematurity, maternal stress and children’s intelligence quotient at age 11: A longitudinal study. Sci. Rep..

[57] Largo R.H., Pfister D., Molinari L., Kundu S., Lipp A., Due G. (2008). Significance of prenatal, perinatal and postnatal factors in the development of AGA preterm infants at five to seven years. Dev. Med. Child Neurol..

[58] McGauhey P.J., Starfield B., Alexander C., Ensminger M.E. (1991). Social environment and vulnerability of low birth weight children: A social-epidemiological perspective. Pediatrics.

[59] Habersaat S., Pierrehumbert B., Forcada-Guex M., Nessi J., Ansermet F., Müller-Nix C., Borghini A. (2014). Early stress exposure and later cortisol regulation: Impact of early intervention on mother–infant relationship in preterm infants. Psychol. Trauma: Theory Res. Pract. Policy.

[60] Scheiner A.P., Sexton M.E. (1991). Prediction of developmental outcome using a perinatal risk inventory. Pediatrics.

[61] Pierrehumbert B., Borghini A., Forcada-Guex M., Jaunin L., Müller-Nix C., Ansermet F. (2004). Validation française d’un questionnaire de stress post-traumatique destiné aux parents d’enfants présentant un risque périnatal élevé. Ann. Médico-Psychol. Rev. Psychiatr..

[62] Achenbach T.M., Edelbrock C. (1991). Child behavior checklist. Burlington.

[63] Pierrehumbert B., Ramstein T., Karmaniola A., Halfon O. (1996). Child care in the preschool years: Attachment, behaviour problems and cognitive development. Eur. J. Psychol. Educ..

[64] Dressendörfer R.A., Kirschbaum C., Rohde W., Stahl F., Strasburger C.J. (1992). Synthesis of a cortisol-biotin conjugate and evaluation as a tracer in an immunoassay for salivary cortisol measurement. J. Steroid Biochem. Mol. Biol..

[65] Pruessner J.C., Kirschbaum C., Meinlschmid G., Hellhammer D.H. (2003). Two formulas for computation of the area under the curve represent measures of total hormone concentration versus time-dependent change. Psychoneuroendocrinology.

[66] Braaten E.B. (2018). Child Behavior Checklist. The SAGE Encyclopedia of Intellectual and Developmental Disorders.

[67] Miller R., Stalder T., Jarczok M., Almeida D.M., Badrick E., Bartels M., Boomsma D.I., Coe C.L., Dekker M.C.J., Donzella B. (2016). The CIRCORT database: Reference ranges and seasonal changes in diurnal salivary cortisol derived from a meta-dataset comprised of 15 field studies. Psychoneuroendocrinology.

[68] Granger D.A., Serbin L.A., Schwartzman A., Lehoux P., Cooperman J., Ikeda S. (1998). Children’s Salivary Cortisol, Internalising Behaviour Problems, and Family Environment: Results from the Concordia Longitudinal Risk Project. Int. J. Behav. Dev..

[69] Oosterlaan J., Geurts H.M., Knol D.L., Sergeant J.A. (2005). Low basal salivary cortisol is associated with teacher-reported symptoms of conduct disorder. Psychiatry Res..

[70] Shirtcliff E.A., Granger D.A., Booth A., Johnson D. (2005). Low salivary cortisol levels and externalizing behavior problems in youth. Dev. Psychopathol..

[71] De Graaf J., Van Den Akker E.L.T., Van Lingen R.A., Groot Jebbink L.J.M., De Jong F.H., Grunau R.E., Van Dijk M., Tibboel D. (2014). Five-year follow-up of effects of neonatal intensive care and morphine infusion during mechanical ventilation on diurnal cortisol rhythm. J. Pediatr..

[72] Ladd C.O., Huot R.L., Thrivikraman K.V., Nemeroff C.B., Meaney M.J., Plotsky P.M. (2000). Long-term behavioral and neuroendocrine adaptations to adverse early experience. Prog. Brain Res..

[73] Pryce C.R., Feldon J. (2003). Long-term neurobehavioural impact of the postnatal environment in rats: Manipulations, effects and mediating mechanisms. Neurosci. Biobehav. Rev..

[74] Andersen S.L. (2003). Trajectories of brain development: Point of vulnerability or window of opportunity?. Neurosci. Biobehav. Rev..

[75] Brummelte S., Grunau R.E., Chau V., Poskitt K.J., Brant R., Vinall J., Gover A., Synnes A.R., Miller S.P. (2012). Procedural pain and brain development in premature newborns. Ann. Neurol..

[76] Duerden E.G., Grunau R.E., Guo T., Foong J., Pearson A., Au-Young S., Lavoie R., Chakravarty M.M., Chau V., Synnes A. (2018). Early Procedural Pain Is Associated with Regionally-Specific Alterations in Thalamic Development in Preterm Neonates. J. Neurosci..

[77] Clark C.A.C., Woodward L.J., Horwood L.J., Moor S. (2008). Development of Emotional and Behavioral Regulation in Children Born Extremely Preterm and Very Preterm: Biological and Social Influences. Child Dev..

[78] Wadsby M., Nelson N., Ingemansson F., Samuelsson S., Leijon I. (2014). Behaviour problems and cortisol levels in very-low-birth-weight children. Nord. J. Psychiatry.

[79] Loe I.M., Lee E.S., Luna B., Feldman H.M. (2011). Behavior problems of 9–16year old preterm children: Biological, sociodemographic, and intellectual contributions. Early Hum. Dev..

[80] Elgen I., Sommerfelt K., Markestad T. (2002). Population based, controlled study of behavioural problems and psychiatric disorders in low birthweight children at 11 years of age. Arch. Dis. Child. Fetal Neonatal Ed..

[81] Bartal T., Adams M., Natalucci G., Borradori-Tolsa C., Latal B. (2020). Behavioral problems in very preterm children at five years of age using the Strengths and Difficulties Questionnaire: A multicenter cohort study. Early Hum. Dev..

[82] Schechter D.S., Suardi F., Manini A., Cordero M.I., Rossignol A.S., Merminod G., Gex-Fabry M., Moser D.A., Serpa S.R. (2015). How do maternal PTSD and alexithymia interact to impact maternal behavior?. Child Psychiatry Hum. Dev..

[83] Muller-Nix C., Forcada-Guex M., Pierrehumbert B., Jaunin L., Borghini A., Ansermet F. (2004). Prematurity, maternal stress and mother-child interactions. Early Hum. Dev..

[84] Giddens A., Bowlby J. (1970). Attachment and Loss, Volume I: Attachment. Br. J. Sociol..

[85] Gondwe K.W., Holditch-Davis D. (2015). Posttraumatic stress symptoms in mothers of preterm infants. Int. J. Afr. Nurs. Sci..

[86] Sullivan R., Perry R., Sloan A., Kleinhaus K., Burtchen N. (2011). Infant Bonding and Attachment to the Caregiver: Insights from Basic and Clinical Science. Clin. Perinatol..

[87] Spittle A., Orton J., Anderson P.J., Boyd R., Doyle L.W. (2015). Early developmental intervention programmes provided post hospital discharge to prevent motor and cognitive impairment in preterm infants. Cochrane Database Syst. Rev..

[88] Landry S.H., Smith K.E., Swank P.R. (2006). Responsive parenting: Establishing early foundations for social, communication, and independent problem-solving skills. Dev. Psychol..

[89] Landsem I.P., Handegård B.H., Ulvund S.E., Kaaresen P.I., Rønning J.A. (2015). Early intervention influences positively quality of life as reported by prematurely born children at age nine and their parents: A randomized clinical trial. Health Qual. Life Outcomes.

[90] McKenzie-McHarg K., Ayers S., Ford E., Horsch A., Jomeen J., Sawyer A., Stramrood C., Thomson G., Slade P. (2015). Post-traumatic stress disorder following childbirth: An update of current issues and recommendations for future research. J. Reprod. Infant Psychol..

[91] Als H. (1986). A Synactive Model of Neonatal Behavioral Organization. Phys. Occup. Ther. Pediatr..

[92] Cho E.-S., Kim S.-J., Kwon M.S., Cho H., Kim E.H., Jun E.M., Lee S. (2016). The Effects of Kangaroo Care in the Neonatal Intensive Care Unit on the Physiological Functions of Preterm Infants, Maternal–Infant Attachment, and Maternal Stress. J. Pediatric Nurs..

[93] Lee J., Bang K.-S. (2011). The Effects of Kangaroo Care on Maternal Self-esteem and Premature Infants’ Physiological Stability. Korean J. Women Health Nurs..

[94] Gitau R., Modi N., Gianakoulopoulos X., Bond C., Glover V., Stevenson J. (2002). Acute effects of maternal skin-to-skin contact and massage on saliva cortisol in preterm babies. J. Reprod. Infant Psychol..

[95] Mörelius E., Örtenstrand A., Theodorsson E., Frostell A. (2015). A randomised trial of continuous skin-to-skin contact after preterm birth and the effects on salivary cortisol, parental stress, depression, and breastfeeding. Early Hum. Dev..

[96] Milgrom J., Newnham C., Anderson P.J., Doyle L.W., Gemmill A.W., Lee K., Hunt R.W., Bear M., Inder T. (2010). Early Sensitivity Training for Parents of Preterm Infants: Impact on the Developing Brain. Pediatric Res..

[97] Athanasopoulou E., Fox J.R.E. (2014). Effect of kangaroo mother care on maternal mood and interaction patterns between parents and their preterm, low birth weight infants: A systematic review. Infant Ment. Health J..

[98] Bullinger A., Goubet N. (1999). Le bébé prématuré, acteur de son développement. Enfance.

[99] White-Traut R.C., Schwertz D., McFarlin B., Kogan J. (2009). Salivary cortisol and behavioral state responses of healthy newborn infants to tactile-only and multisensory interventions. J. Obstet. Gynecol. Neonatal Nurs. JOGNN.

